# Aspartame and Its Metabolites Cause Oxidative Stress and Mitochondrial and Lipid Alterations in SH-SY5Y Cells

**DOI:** 10.3390/nu15061467

**Published:** 2023-03-18

**Authors:** Lea Victoria Griebsch, Elena Leoni Theiss, Daniel Janitschke, Vincent Konrad Johannes Erhardt, Tobias Erhardt, Elodie Christiane Haas, Konstantin Nicolas Kuppler, Juliane Radermacher, Oliver Walzer, Anna Andrea Lauer, Veronika Matschke, Tobias Hartmann, Marcus Otto Walter Grimm, Heike Sabine Grimm

**Affiliations:** 1 Experimental Neurology, Saarland University, 66424 Homburg, Germany; 2 Physical Therapy, Campus Karlsruhe, SRH University of Applied Health Sciences, 76185 Karlsruhe, Germany; 3 Nutrition Therapy and Counseling, Campus Rheinland, SRH University of Applied Health Sciences, 51377 Leverkusen, Germany; 4 Department of Cytology, Institute of Anatomy, Medical Faculty, Ruhr University Bochum, 44801 Bochum, Germany; 5 Deutsches Institut für DemenzPrävention, Saarland University, 66424 Homburg, Germany

**Keywords:** synthetic sweetener, lipids, lipid droplets, phosphatidylcholine, phosphatidylethanolamine, ROS, mitochondrial damage

## Abstract

Due to a worldwide increase in obesity and metabolic disorders such as type 2 diabetes, synthetic sweeteners such as aspartame are frequently used to substitute sugar in the diet. Possible uncertainties regarding aspartame’s ability to induce oxidative stress, amongst others, has led to the recommendation of a daily maximum dose of 40 to 50 mg per kg. To date, little is known about the effects of this non-nutritive sweetener on cellular lipid homeostasis, which, besides elevated oxidative stress, plays an important role in the pathogenesis of various diseases, including neurodegenerative diseases such as Alzheimer’s disease. In the present study, treatment of the human neuroblastoma cell line SH-SY5Y with aspartame (271.7 µM) or its three metabolites (aspartic acid, phenylalanine, and methanol (271.7 µM)), generated after digestion of aspartame in the human intestinal tract, resulted in significantly elevated oxidative stress associated with mitochondrial damage, which was illustrated with reduced cardiolipin levels, increased gene expression of SOD1/2, PINK1, and FIS1, and an increase in APF fluorescence. In addition, treatment of SH-SY5Y cells with aspartame or aspartame metabolites led to a significant increase in triacylglycerides and phospholipids, especially phosphatidylcholines and phosphatidylethanolamines, accompanied by an accumulation of lipid droplets inside neuronal cells. Due to these lipid-mediating properties, the use of aspartame as a sugar substitute should be reconsidered and the effects of aspartame on the brain metabolism should be addressed in vivo.

## 1. Introduction

In recent decades, the medical and socio-economic interest in non-nutritive and synthetic sweeteners has risen due to a worldwide increase in obesity and metabolic diseases such as type 2 diabetes, among other things [1,2]. These diseases are known to be caused by the so-called “Western Diet”, containing high contents of refined carbohydrates and saturated fats, and are risk factors for cardiovascular diseases, which is the number one disease worldwide [3].

Non-nutritive sweeteners (NNS) are perceived to be hundreds of times sweeter than conventional carbohydrate sugar (sucrose). Therefore, they are added to foods in small amounts and have a very low calorie count [4]. A popular non-nutritive and synthetic sweetener found in many “low-sugar” or “diet” foods is the dipeptide aspartame (*N*-L-α-aspartyl-L-phenylalanine-1-methyl ester), which tastes 200 times sweeter than the same amount of conventional household sugar (sucrose) [5].

Aspartame has been controversially discussed as a potentially toxic agent. In view of possible uncertainties and controversies about the safety of aspartame, the US Food and Drug Administration (FDA) and the European Food Safety Authority (EFSA) have made recommendations of a daily limit of 50 and 40 mg per kilogram of body weight, respectively [6].

Besides the effects which indicate that aspartame is involved in an increased risk of cancers such as leukemia [7], there have been studies carried out which have examined its impact on the brain [8]. The consumption of aspartame has been found to elevate the levels of phenylalanine and aspartic acid in the brain. Phenylalanine blocks the transport of important amino acids to the brain, contributing to reduced levels of dopamine and serotonin, whereas aspartic acid at high concentrations causes hyperexcitability of neurons [9]. Furthermore, aspartame consumption has been found to impair the memory of rats [10], as well as to influence the behavior of mice in terms of cognitive responses and learning [11].

Based on various in vitro and in vivo studies, aspartame is known to be responsible for an increase in cellular oxidative stress in the brain [12,13]. Oxidative stress occurs when there is an imbalance between the production of reactive oxygen species (ROS) and their cellular antioxidant compensatory mechanisms/systems. ROS such as hydroxyl radicals, hydroperoxyl radicals, or superoxide anions are molecules that contain highly reactive oxygen because they are in possession of extra electrons. This can lead to damage of other cellular subcomponents, namely certain amino acids, proteins, DNA, or lipids. In recent decades, there has been increasing evidence that oxidative stress is closely linked to lipid metabolism in cells. Hydroperoxyl radicals are known to cause lipid peroxidation, as they attack carbon double bonds that are particularly found in polyunsaturated fatty acids (PUFAs) [14]. Neuronal cells are composed of a high proportion of PUFAs, which makes them particularly susceptible to ROS and lipid peroxidation [15]. The possible consequences range from reduced acetylcholinesterase activity [16] to memory impairment or impaired emotional behavior in rats [17].

Furthermore, ROS are known to be involved in the pathogenesis of several metabolic, but also neurodegenerative, diseases [18]. For example, Alzheimer’s disease is the most common form of dementia [19] and is clinically characterized by a progressive loss of cognitive functions, such as a decrease in the ability to remember new data, but also by changes in the patient’s behavior [20]. Pathomechanistic features include an extracellular accumulation of Aβ-peptide and intracellular phosphorylated tau fibrils in various brain regions, such as the hippocampus, which is particularly important for memory formation [21]. In addition, Alzheimer’s disease is mediated by mitochondrial dysfunction with elevated ROS levels [22], which have been shown to promote Aβ accumulation and tau phosphorylation [23].

To date, little is known about the influence of aspartame consumption on neuronal lipid metabolism, particularly in relation to oxidative stress. Therefore, we investigated whether aspartame treatment affects cellular lipid levels, oxidative stress, and gene expressions of several related enzymes in the neuronal cell line SH-SY5Y.

## 2. Materials and Methods

### 2.1. Chemicals and Reagents

Gibco™ DMEM High Glucose and Gibco™ DMEM No Glucose cell culture media were purchased from Life Technologies Europe (Bleiswijk, The Netherlands) via Merck (Darmstadt, Germany). HyClone ™ Fetal Bovine Serum was acquired from GE Healthcare Life Scienes (Logan, UT, USA). MEM Non-essential Amino Acid Solution was supplied by Sigma^®^ Life Science (Gillingham, UK). D (+)-Glucose was supplied by Carl Roth^®^ (Karlsruhe, Germany). Aspartame was purchased from Sigma Aldrich^®^ (St. Louis, MO, USA). L-aspartic acid and L-phenylalanine were acquired from Alfa Aesar by Thermo Fisher Scientific (Kandel, Germany). Methanol and high quality HPLC water were purchased from Fisher Chemicals^®^ (Loughborough, UK).

### 2.2. Cell Culture and Treatment

Human neuroblastoma cells (SH-SY5Y) were cultured in Gibco™ DMEM High Glucose containing 4 g/L D-glucose, 10% fetal bovine serum, and 1% non-essential amino acid solution at 37.0 °C and 5% CO_2_.

At 90% confluency, cells were seeded in 6-well plates (Falcon^®^ Multiwell 6 well from Corning Incorporated, Corning, NY, USA) to perform the incubations described below. At a confluence of about 80% in the 6-well plate, the FBS in the DMEM was reduced to 2.5% for 16 h, followed by the first incubation for 24 h. Control cells were incubated with culture medium alone, and aspartame and its metabolites were directly solved in culture medium. Treatment consisted of either controls or aspartame or metabolites, which is based on the fact that aspartame is enzymatically cleaved into its three metabolites by gastrointestinal enzymes after ingestion [24]. However, the complete molecule can also possibly cross the endothelium under certain conditions, making it reasonable to investigate the effects of the aspartame molecule as well.

Control-treated cells were incubated with DMEM No Glucose containing 1 g/L sterile filtered D-glucose, 2.5% FBS, and 1% NEAA. Aspartame-treated cells were incubated with aspartame at a molarity of 271.7 µM, which was dissolved in DMEM No Glucose containing 1 g/L sterile filtered D-glucose, 2.5% FBS, and 1% NEAA. Metabolite-treated cells were incubated with DMEM No Glucose containing 1 g/L sterile filtered D-glucose, 2.5% FBS, 1% NEAA, and the combination of L-aspartic acid, L-phenylalanine, and methanol, each with a molarity of 271.7 µM (see Figure S1). The aim of the experiment was to use the same number of molecules for the metabolites and for aspartame. Therefore, we decided to choose the same molarity of the metabolites compared to aspartame. Since one molecule of aspartame breaks down into one molecule of aspartic acid, one molecule of phenylalanine, and one molecule of methanol, a concentration of 271.7 µM was therefore used for all metabolites as well as aspartame. Since the molar mass of the metabolites and aspartame are different, it follows from this consideration that a different amount of metabolites and aspartame must be used. Therefore, 36 µg/L aspartic acid, 45 µg/L phenylalanine, and 9 µg/L methanol were used according to the molar mass. The dose was defined by applying the recommended daily maximum intake of aspartame at the blood distribution volume of an average weighted person, as well as by using the existing literature [25]. After 24 h, the medium was replaced by the same medium containing aspartame or the three metabolites for another 24 h, resulting in a total treatment time of 48 h, which is in line with the literature [26,27] and enabled sufficient time for potential effects to be measured afterwards.

### 2.3. Transmission Electron Microscopy

Control-, aspartame-, or metabolite-treated cells were plated in T25 cell culture flasks and cultured to 100% confluence as described above. Afterwards, the cells were fixed with 2% formaldehyde (#15714-S, Electron Microscopy Sciences, Hatfield, PA, USA), 2.5% glutaraldehyde (#G5882, Merck, Darmstadt, Germany), and 2 mM CaCl_2_ in 0.15 mM cacodylate buffer. The samples were then stained with 1% osmium tetroxide in H_2_O for 1 h. Cells were detached from the bottom of the flask using cell scrapers, centrifuged at 1800 rpm for 3 min, and the cell bands were embedded in agar-agar (3% in PB). Samples were dehydrated in an ascending ethanol series starting with 50% ethanol followed by incubation in 70% ethanol, 1% uranyl acetate (#21447, Polyscience Europe, Heidelberg, Germany), and 1% phosphotungstic acid (#455970, Merck, Darmstadt, Germany) overnight at 4 °C. The next day, dehydration was continued with an ascending ethanol series (80–100%). The preparations were carefully transferred into epoxy resin. For this purpose, the tissue was first incubated in propylene oxide (#807027, Merck, Germany), followed by an ascending series of propylene oxide and EPON mixtures. This embedding procedure started with propylene oxide/EPON in a 3:1 ratio, followed by a 1:1 ratio, and ended with a 1:3 ratio. Finally, the samples were permeated with pure EPON overnight at 20 °C. On the third day of embedding, the EPON was renewed. The EPON-embedded samples were polymerized at 60 °C for two days. EPON consists of glycidyl ether (#21045.02, Serva, Heidelberg, Germany), methyl nadic anhydride (#29452.02, Serva, Heidelberg, Germany), 2-dodecenyl succinic anhydride (#20755.01, Serva, Heidelberg, Germany), and 2,4,6-tris(dimethylaminomethyl)phenol (#36975.01, Serva, Heidelberg, Germany) in a 5.4:3.8:1.84:1 mixture. Ultrathin slices (70 nm) were cut with an Ultracut E Reichert-Jung (Leica Microsystems GmbH, Wetzlar, Germany) using a DiATOME histo diamond knife (45°, 6 mm, MX559; Diatome AG, Nidau, Switzerland). Images were taken with a Zeiss LEO 910 transmission electron microscope equipped with a digital camera.

### 2.4. Detection of Cardiolipin

In order to measure the cardiolipin levels via fluorescence, a Cardiolipin Assay Kit from Abcam (ab241036, Berlin, Germany) was utilized according to the manufacturer’s protocol.

### 2.5. Detection of Oxidative Stress

Aminophenyl fluorescin (APF) from Thermo Fisher Scientific (A36003) was used to measure oxidative stress markers by fluorescence. After completion of incubation, adherent cells were washed with a Life Cell Imaging Solution (LCIS) containing sodium chloride (140 mM), potassium chloride (2.5 mM), and calcium chloride (1.8 mM). The cells were then exposed to 10 μM APF resolution (in LCIS) and the fluorescence was measured at an excitation wavelength of 490 nm and an emission wavelength of 515 nm.

### 2.6. Measurement of Lipid Species via Mass Spectrometry

#### 2.6.1. Sample Preparation

After the incubation period, cells were harvested on ice, the incubation medium was removed, the cells were washed with HPLC water, and then they were scraped into a volume of 50 µL HPLC water per well. The suspension was transferred to Minilys tubes (neoLab^®^, Heidelberg, Germany) containing Precellys^®^ 1.4 mm zirconia beads and homogenized with Bertin Minilys^®^ (Peqlab, Erlangen, Germany) for 60 s at a speed of 5000/min. A Smith’s bicinchoninic acid assay [28] was performed to measure the protein concentration in the cell homogenates, followed by adjustment of the cell homogenates to a protein concentration of 4 mg/mL in HPLC water.

#### 2.6.2. Lipid Extraction

To ensure mass spectrometric quantification of different lipid species, namely different phospholipids (phosphatidylcholine species and phosphatidylethanolamine species), sphingomyelin, carnitine, and triacylglycerides, a solid/lipid extraction method was performed beforehand. First, a 96-well filter plate (MultiScreen^®^ Solvinert from Merck Millipore, Carrigtwohill, Ireland) was attached to a 96-well deepwell plate (Thermo Fisher Scientific^®^, Rochester, NY, USA). A 6 mm diameter Whatman filter paper (GE Healthcare, Buckinghamshire, UK) was placed in each well of the filter plate, followed by the addition of 6 µL of internal standard (see Table 1) and 25 µL of each adjusted sample. The plate was dehumidified for 60 min under a nitrogen flow. An amount of 150 µL phenyl isothiocyanate (Merck^®^, Darmstadt, Germany) was added to a solution containing 950 µL each of ethanol (Fisher Chemicals^®^, Loughborough, UK), HPLC water, and pyridine (Sigma Aldrich^®^, St. Louis, MO, USA). An amount of 20 µL of this mixture was added to each sample and the plate was incubated at room temperature for 20 min and afterwards was dried for 45 min under a nitrogen flow. Afterwards, to each sample, 300 µL of a mixture of 19 mg ammonium acetate dissolved in 50 mL HPLC methanol (both Fisher Chemicals^®^, Loughborough, UK) was added. The plate was then placed on a plate shaker at 450 rpm for 30 min, followed by centrifugation at 500 rpm for two minutes. The filter plate was removed and 600 µL of a mixture of 5 mM ammonium acetate in methanol/water (97:3, *v*/*v*) was added to each sample. The deep well plate was covered with a silicone mat (Thermo Fisher Scientific^®^, Rochester, NY, USA) and placed on the plate shaker (450 rpm) for a further two minutes before a mass spectrometric analysis could take place.

#### 2.6.3. Mass Spectrometry

A 4000-quadropole linear ion trap (QTrap) with a turbo spray ion source (AB Sciex, Darmstadt, Germany) was used for the detection of the different lipid species. Using the Agilent 1200 HPLC autosampler (Agilent, Böblingen, Germany), each sample was measured as a technical duplicate and subsequently analyzed using Analyst 1.4.2. software (AB SCIEX, Darmstadt, Germany) (see a detailed description in [29]). The detection of different lipid species was carried out using multi-reaction monitoring (MRM) as a scan type with specific parameters (see Table 2) and a measurement time of three minutes for each sample.

### 2.7. Analysis of Gene Expression

To compare the expression of different genes after treatment with aspartame or metabolites, total cellular RNA was extracted with TRIzol reagent according to the manufacturer’s protocol (Thermo Fisher Scientific^®^, Rochester, NY, USA). A Nano Drop 2000 (Thermo Fisher Scientific^®^, Rochester, NY, USA) was used to analyze the concentration and purity of the isolated RNA. All samples were adjusted to an amount of 2 µg RNA and transcribed into complementary DNA (cDNA) using a High-Capacity cDNA Reverse Transcription Kit (Thermo Fisher Scientific^®^, Rochester, NY, USA). A quantitative real-time polymerase chain reaction (RT-PCR) was performed using a Fast SYBR Green (Applied Biosystems, Foster City, CA, USA) and a PikoReal Real-Time PCR System (Thermo Fisher Scientific^®^, Rochester, NY, USA). The changes in expression of the different genes (see used forward and reverse primers listed in Table 3) were measured using the 2^−∆∆Cq^ method after normalization to the most stable housekeeping gene, *HPRT* (stability value = 0.061). Genes of interest included those related to oxidative stress (*SOD1* and *SOD2*), mitophagy (*PINK1*), and mitochondrial fission (*FIS1*).

### 2.8. Detection of Cell Viability

To identify potential cytotoxic effects on SH-SY5Y cells, the lactate dehydrogenase (LDH) activity was measured by utilizing a Cytotoxicity Detection KitPLUS (Roche Diagnostics, Mannheim, Germany), following the manufacturer’s protocol. The cytotoxicity was below 5% under all incubation conditions.

### 2.9. Data and Statistical Analysis

For each sample, the counts per second of each lipid, extracted using the Analyst 1.4.2 Software from AB Sciex, were normalized to the specific internal lipid class standard. Afterwards, the means of two technical lipid/standard ratio duplicates were calculated. The relative changes between aspartame-treated, metabolite-treated, or control-treated cell samples (set to 100%) were calculated and plotted as percentages in bar graphs. To analyze the statistical significance of the observed changes, an ANOVA followed by a Tukey HSD post hoc test was performed. These calculations were performed with “R” (R Core Team 2020; Vienna, Austria; https://www.R-project.org/; accessed on 1 June 2021). Significance levels were set at * *p* ≤ 0.05, ** *p* ≤ 0.01, and *** *p* ≤ 0.001.

In the volcano plots, the relative change in each lipid type was plotted on the *y*-axis (represented as −Log10 of percent fold change) against the corresponding *p*-value (represented as Log2; calculated by the two-tailed Student’s *t*-test) on the *x*-axis. The mean standard error of the mean (SEM) was calculated as the mean SEM of the cells treated with aspartame and metabolites and is represented by two vertical lines. Volcano plots were generated using the R package “EnhancedVolcano” (Kevin Blighe, Sharmila Rana and Myles Lewis (2020), version 1.6.0., https://github.com/kevinblighe/EnhancedVolcano; accessed on 1 June 2021).

The statistical significance of the observed shifts in down- and up-regulated parameters within aspartame- or metabolite-treated cells was calculated using binomial test. To examine the significant differences in lipid distribution between aspartame- and metabolite-treated cells, a Fisher’s exact test was used.

## 3. Results

The aim of this study was to analyze the influence of aspartame as well as its metabolites on oxidative stress and lipid homeostasis of human neuroblastoma cells. For this purpose, SH-SY5Y cells were incubated either with aspartame as a complete molecule or with its three metabolites at equimolar concentrations over a period of 24 + 24 h (48 h in total). The separate incubation with the three aspartame metabolites was performed because aspartame has been reported to be metabolized into the three metabolites, i.e., aspartic acid (40%), phenylalanine (50%), and methanol (10%), after digestion and to make this in vitro study as representative as possible of aspartame intake in humans.

### 3.1. Transmission Electron Microcopy

**Figure 1:**
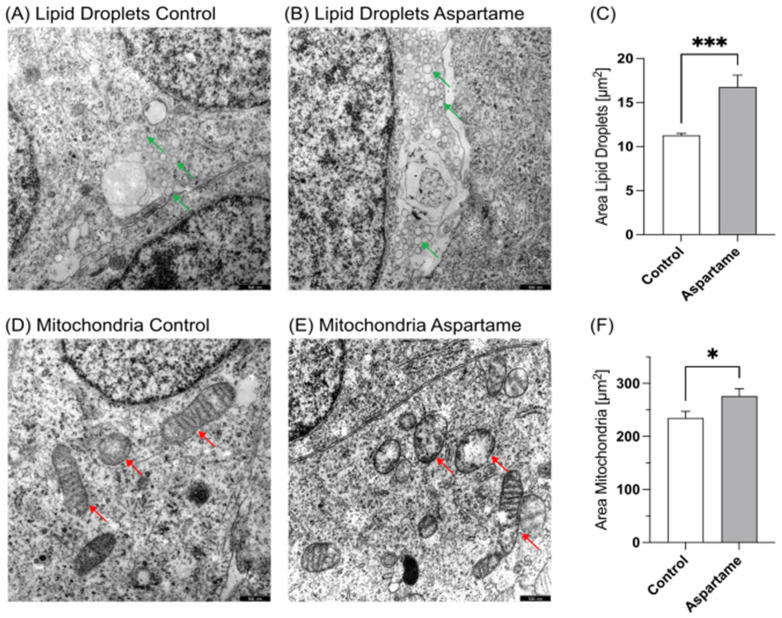
Transmission electron microscopy of SH-SY5Y wild-type cells. (**A**) Control-treated SH-SY5Y cells at 12,000× magnification. Green arrows mark lipid droplets. (**B**) SH-SY5Y cells treated with aspartame at 12,000× magnification. Green arrows mark lipid droplets. (**C**) Comparison of the area of lipid droplets in control- and aspartame-treated cells in a bar chart. Error bars represent the standard error of the mean (SEM). The statistical significance was set as *** *p* ≤ 0.001. (**D**) Control-treated SH-SY5Y cells at 12,000× magnification. Red arrows show mitochondria. (**E**) SH-SY5Y cells treated with aspartame at 12,000× magnification. Red arrows show mitochondria. (**F**) Comparison of the area of mitochondria in control- and aspartame-treated cells. Error bars represent the standard error of the mean (SEM). The statistical significance was set as * *p* ≤ 0.05. Scale bars for (**A**,**B**,**D**,**E**): 500 nm.

**Figure 2:**
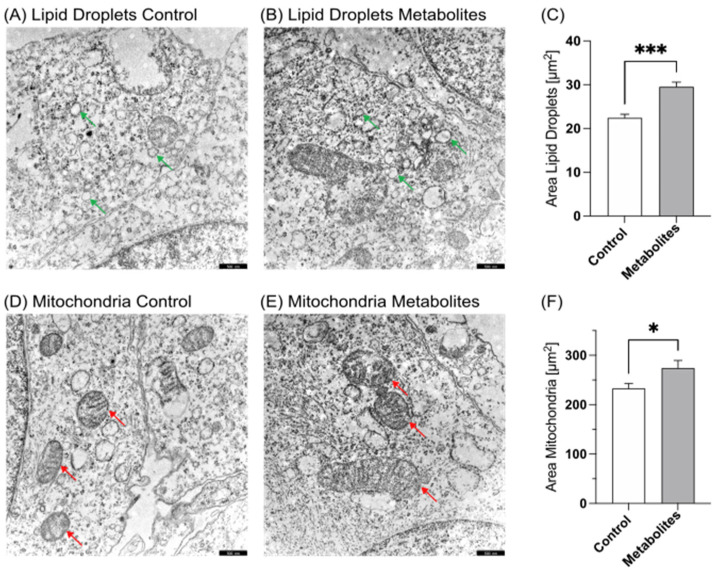
Transmission electron microscopy of SH-SY5Y wild-type cells. (**A**) Control-treated SH-SY5Y cells at 12,000× magnification. Green arrows mark lipid droplets. (**B**) SH-SY5Y cells treated with aspartame metabolites at 12,000× magnification. Green arrows mark lipid droplets. (**C**) Comparison of the area of lipid droplets in control- and aspartame metabolite-treated cells in a bar chart. Error bars represent the standard error of the mean (SEM). The statistical significance was set as *** *p* ≤ 0.001. (**D**) Control-treated SH-SY5Y cells at 12,000× magnification. Red arrows show mitochondria. (**E**) SH-SY5Y cells treated with aspartame metabolites at 12,000× magnification. Red arrows show mitochondria. (**F**) Comparison of the area of mitochondria in control- and aspartame metabolite-treated cells. Error bars represent the standard error of the mean (SEM). The statistical significance was set as * *p* ≤ 0.05. Scale bars for (**A**,**B**,**D**,**E**): 500 nm.

Incubated and fixated cells were examined via transmission electron microscopy to determine the possible cell morphological effects of aspartame. As shown in Figure 1A–C and Figure 2A–C, a significant accumulation of lipid droplets (LD) was observed in aspartame- or metabolite-treated cells compared to control-treated cells. Significant differences could be detected between the amount as well as the area of lipid droplets; aspartame treatment resulted in a significant increase in the mean LD area to 145.8% (*p* ≤ 0.001), while treatment with the metabolites resulted in a significant increase to 131.7% (*p* ≤ 0.001) compared to control-treated cells. Moreover, we found the structural mitochondrial integrity to be impaired after both aspartame and metabolite treatment (Figure 1D,E and Figure 2D,E). The resulting mean mitochondrial area accounted for 123.0% (*p* = 0.0187) and 118.8% (*p* = 0.0188), respectively.

### 3.2. Analysis of Mitochondrial Damage

**Figure 3:**
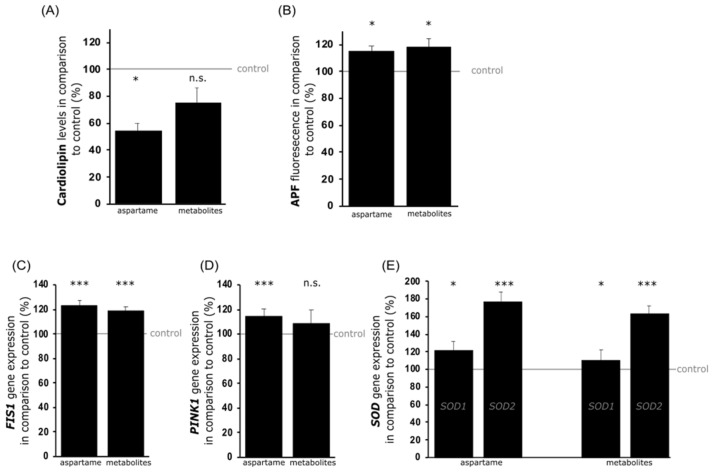
Effects of aspartame or aspartame metabolites on mitochondrial integrity and cellular oxidative stress. Neuroblastoma cells (SH-SY5Y) were treated with control, aspartame, or aspartame metabolites as described before. Afterwards, the levels of cardiolipin (**A**) and APF (**B**), as well as gene expression levels of *FIS1* (**C**), *PINK1* (**D**), *SOD1*, and *SOD2* (**E**), were detected. A comparison of the corresponding changes in aspartame- and metabolite-treated SH-SY5Y cells is illustrated by bar charts. Error bars represent the standard error of the mean (SEM) and the statistical significance was set as * *p* ≤ 0.05 and *** *p* ≤ 0.001 (n.s.: not significant).

To specify the findings of potentially impaired mitochondria, a cardiolipin assay was performed. Cardiolipin is a glycerophosphate consisting of a glycerol backbone linked to two phosphatide groups, each esterified with two fatty acids. It is considered a major component of the inner and outer mitochondrial membrane and is required for oxidative phosphorylation as it stabilizes respiratory chain supermolecules [30]. Accumulation of ROS and mitochondrial damage lead to loss of cardiolipin [31]. The results from the fluorescence-dependent cardiolipin assay showed that cardiolipin levels were significantly reduced to 56.7% ± 5.6% (*p* = 0.011) after aspartame treatment or not significantly downregulated to 77.9% ± 11.8% (*p* = 0.104) in metabolite-treated SH-SY5Y cells (see Figure 3A). To further examine this potential mitochondrial damage due to aspartame treatment, the gene expression of mitochondrial fission 1 (*FIS1*) was analyzed. FIS1 is a protein localized mainly in the outer mitochondrial membrane and is responsible for mitochondrial fission as a response to mitochondrial damage [32]. Treatment with aspartame resulted in a significant increase in *FIS1* expression to 123.5% (*p* ≤ 0.001) in SH-SY5Y cells. Similarly, in metabolite-treated cells, a significant upregulation of *FIS1* transcription to 118.6% (*p* ≤ 0.001) was detected (see Figure 3C).

Mitochondrial damage activates so-called “mitophagy”. This is a cellular process in which dysfunctional mitochondria are packaged in a phospholipid monolayer membrane that fuses with the lysosome to form an autolysosome, in which damaged mitochondria are degraded [33]. This process is mediated by various signaling proteins such as *PINK1*. In the present study, the incubation of SH-SY5Y cells with aspartame resulted in a significantly upregulated *PINK1* expression to 117.0% (*p* ≤ 0.001). Similarly, treatment with the aspartame metabolites also resulted in an elevated expression of *PINK1*, but without reaching statistical significance (110.9%; *p* = 0.229) (see Figure 3D).

Moreover, mitochondrial damage is associated with the release of ROS. To determine whether aspartame-induced mitochondrial damage and mitophagy were related to ROS, a 3′-(*p*-aminophenyl) fluorescein (APF) assay was performed. APF is a molecule that produces a fluorescence signal solely due to its reaction with hydroxyl radicals or peroxynitrite anions. Under the incubation conditions present in this study, the fluorescence signal of APF was significantly increased to 114.7% (*p* = 0.013) by aspartame and to 118.2% (*p* = 0.018) due to the metabolites (see Figure 3B). To verify the elevated oxidative stress triggered by the treatment of SH-SY5Y cells with aspartame, the gene expressions of the counter-regulatory antioxidant enzymes superoxide dismutase 1 and 2 (SOD1 and SOD2) were examined, which are expressed in the cytoplasm and the mitochondrial matrix, respectively [34]. In line with the data obtained from the APF assay suggesting increased oxidative stress, aspartame treatment led to a significantly increased expression of *SOD1* to 121.7% (*p* = 0.047) and *SOD2* to 176.9% (*p* < 0.001). Metabolite treatment resulted in a significant increase in *SOD1* expression to 110.3% (*p* = 0.048), as well as of *SOD2* to 163.1% (*p* < 0.001) (see Figure 3E).

### 3.3. Lipid Analysis

As presented above, electron microscopy of aspartame- and metabolite-treated SH-SY5Y cells showed impairments of the mitochondria as well as a significant accumulation of lipid droplets.

To determine more precisely whether aspartame treatment and the resulting oxidative stress affect the lipid metabolism and certain lipid species, a semi-quantitative mass spectrometric analysis was carried out, including more than 300 parameters. In this study, the focus was on (a) phospholipids, which are known to be the most important lipid species in the cell membranes, (b) neutral lipids, such as triacylglycerides (TAG), and (c) carnitines. The data obtained for each lipid species were normalized to the corresponding deuterated standard of the lipid class and calculated as the x-fold change in aspartame- or metabolite-treated cells compared to control-treated cells.

#### 3.3.1. Analysis of Triacylglycerol Species

**Figure 4:**
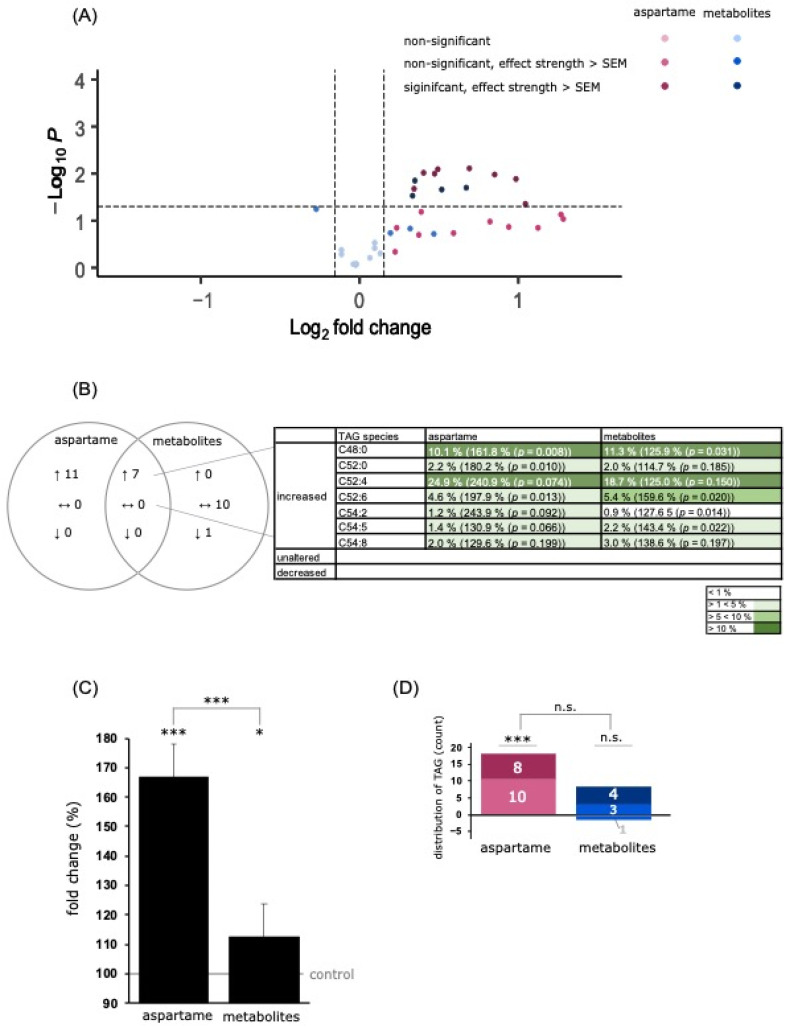
Effects of aspartame or aspartame metabolites on triacylglycerol (TAG) species. Neuroblastoma cells (SH-SY5Y) were treated as described before with control, aspartame, or aspartame metabolites for 48 h and analyzed using a semi-quantitative shotgun lipidomics approach. Each diagram represents the changes in the aspartame- or metabolite-treated cells in comparison to control-treated cells. (**A**) In the volcano plot, each triglyceride species is graphically represented by a pink (aspartame treatment) or blue (aspartame metabolite treatment) dot which is plotted with its fold change (*x*-axis) against its according *p*-value (*y*-axis). Light blue and light pink dots represent no significant changes. Medium blue and medium pink dots represent a fold change which is greater than the mean standard error of the mean (SEM). Dark blue and dark pink dots represent a fold change which is greater than the mean SEM and in addition has a *p*-value smaller than 0.05 (which was defined as the statistical significance level). (**B**) Venn diagram of TAG species after incubation of SH-SY5Y cells with aspartame or aspartame metabolites. Alterations of the same species are visible in the overlapping part. (**C**) The bar chart shows the relative fold change of all measured TAG species after aspartame treatment compared to treatment with aspartame metabolites. Error bars represent the standard error of the mean (SEM), and the statistical significance was set as * *p* ≤ 0.05 and *** *p* ≤ 0.001. (**D**) Distribution of TAG species, classified by the amount of increased or decreased TAG species. Medium blue and medium pink represent a fold change which is greater than the mean standard error of the mean (SEM). Dark blue and dark pink represent a fold change which is greater than the mean SEM and in addition has a *p*-value smaller than 0.05. The applied statistical analyses are described in detail in Section 2.9 (n.s. not significant).

A mass spectrometric analysis revealed a significant increase in the total TAG species to 166.8% ± 19.3% (*p* ≤ 0.001) after aspartame treatment and to 112.4% ± 9.9% (*p* = 0.014) after metabolite treatment. Additionally, the TAG levels differed significantly between the two incubation conditions (*p* ≤ 0.001) (see Figure 4C). Of the 18 measured TAG species, all were increased to more than the SEM after aspartame treatment. Incubation with the metabolites resulted in an elevation to above the SEM for seven species and a decrease to below the SEM for one species, namely C54:4, to 82.9% ± 8.1% (*p* = 0.059) (see Figure 4D). Both conditions resulted in a joint increase in seven TAG species, namely C48:0, C52:0, C52:4, C52:6, C54:2, C54:5, and C54:8, as shown in Figure 4B. These mass spectrometric results are in line with the electron microscopic studies, which showed a significant accumulation of lipid droplets, which are characterized by a phospholipid membrane and an inner core containing neutral lipids in the form of triacylglycerol [35].

#### 3.3.2. Analysis of Phospholipids

Phospholipids are important structural lipids and a major component of the lipid bilayer of the outer cell membrane. Lipid droplets also contain a phospholipid monolayer [36]. A common feature of phospholipids is their amphiphilic structure, with a hydrophilic phosphate head group and a hydrophobic residue containing two fatty acids. The phosphate group is linked to various organic molecules such as choline or ethanolamine. Phosphatidylcholine (PC) and phosphatidylethanolamine (PE) are the two major phospholipids found in lipidomic studies of the human brain [37] and account for about 32.8% and 35.6% of all phospholipids, respectively. In line with this, PC and PE were also found to be the major phospholipids in SH-SY5Y cells [38]. Besides PC and PE species, we examined the levels of phosphatidylserine, which accounts for approximately 16.6% of total cerebral phospholipids [37]. Treatment with aspartame or metabolites resulted in a balanced effect in terms of increased, unchanged, and decreased PS species in SH-SY5Y cells.

##### PC Species

**Figure 5:**
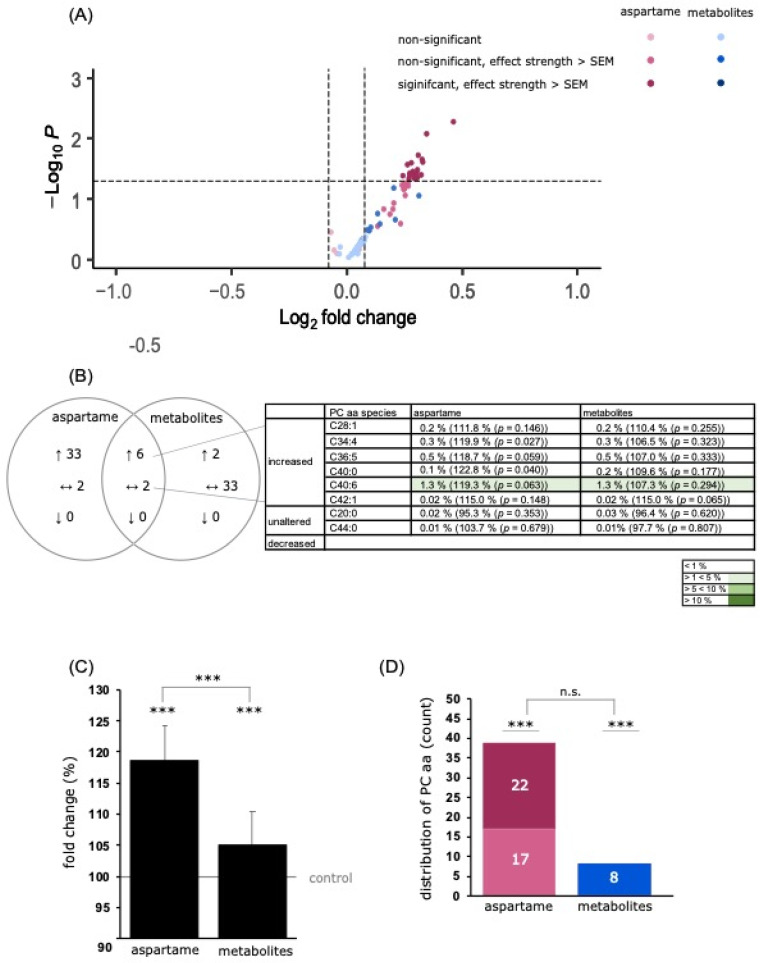
Effects of aspartame or aspartame metabolites on phosphatidylcholine (PCaa) species. Neuroblastoma cells (SH-SY5Y) were treated as described before with control, aspartame, or aspartame metabolites for 48 h and analyzed using a semi-quantitative shotgun lipidomics approach. Each diagram represents the changes in the aspartame- or metabolite-treated cells in comparison to control-treated cells. (**A**) In the volcano plot, each PCaa species is graphically represented by a pink (aspartame treatment) or blue (aspartame metabolite treatment) dot which is plotted with its fold change (*x*-axis) against its according *p*-value (*y*-axis). The volcano plot is structured as the one in Figure 4. (**B**) Venn diagram of PCaa species after incubation of SH-SY5Y cells with aspartame or aspartame metabolites. Alterations of the same species are visible in the overlapping part. (**C**) The bar chart shows the relative fold change of all measured PCaa species after aspartame treatment compared to treatment with aspartame metabolites. Error bars represent the standard error of the mean (SEM), and the statistical significance was set as *** *p* ≤ 0.001. (**D**) Distribution of PCaa species, classified by the amount of increased or decreased PCaa species. The bar chart is structured as the one in Figure 4. The applied statistical analyses are described in detail in Section 2.9 (n.s. not significant).

The mean effect of the analyzed PCaa species was significantly increased to 118.7% ± 8.1% (*p* ≤ 0.001) after aspartame treatment and slightly elevated to 105.0% ± 6.4% (*p* ≤ 0.001) due to the incubation with aspartame metabolites compared to control-treated SH-SY5Y cells. In a direct comparison of aspartame and its metabolites, the effect of aspartame on PCaa species was significantly higher than that of the metabolites (*p* ≤ 0.001) (see Figure 5C). A total of 43 different PCaa species were measured, of which 39 were increased after aspartame treatment and eight after metabolite treatment (see Figure 5D). Six of these species, namely PCaa C28:1, C34:4, C36:5, C40:0, C40:6, and C42:1, were increased under both incubation conditions (see Figure 5D). Interestingly, only three species were found to be slightly decreased after aspartame and two species after metabolite treatment, whereby these effects were within the SEM.

##### PE Species

**Figure 6:**
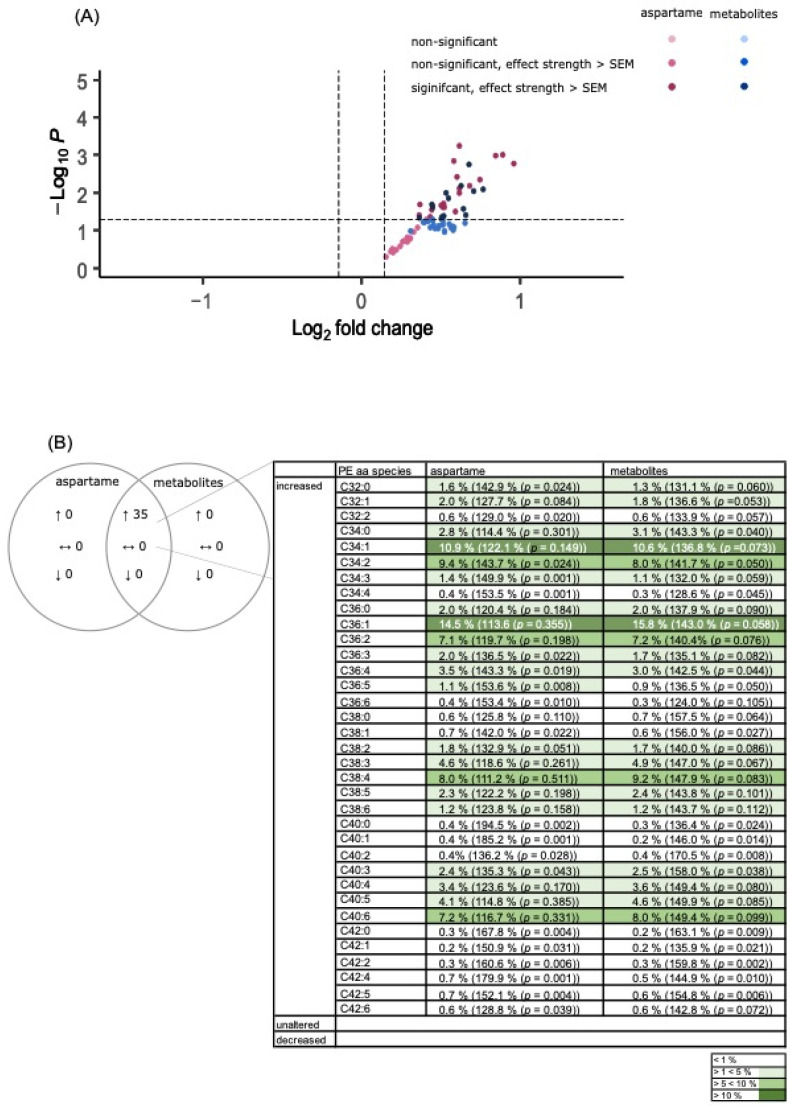

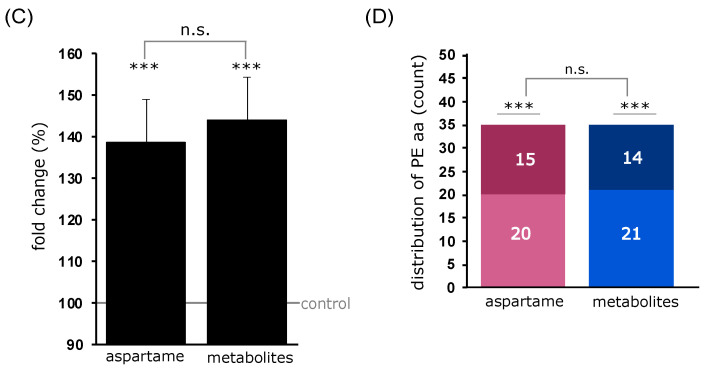
Effects of aspartame or aspartame metabolites on phosphatidylethanolamine (PEaa) species. Neuroblastoma cells (SH-SY5Y) were treated as described before with control, aspartame, or aspartame metabolites for 48 h and analyzed using a semi-quantitative shotgun lipidomics approach. Each diagram represents the changes in the aspartame- or metabolite-treated cells in comparison to control-treated cells. (**A**) In the volcano plot, each PEaa species is graphically represented by a pink (aspartame treatment) or blue (aspartame metabolite treatment) dot which is plotted with its fold change (*x*-axis) against its according *p*-value (*y*-axis). The volcano plot is structured as the one in Figure 4. (**B**) Venn diagram of Peaa species after incubation of SH-SY5Y cells with aspartame or aspartame metabolites. Alterations of the same species are visible in the overlapping part. (**C**) The bar chart shows the relative fold change of all measured Peaa species after aspartame treatment compared to treatment with aspartame metabolites. Error bars represent the standard error of the mean (SEM), and the statistical significance was set as *** *p* ≤ 0.001. (**D**) Distribution of PEaa species, classified by the amount of increased or decreased PEaa species. The bar chart is structured as the one in Figure 4. The applied statistical analyses are described in detail in Section 2.9 (n.s. not significant).

Incubation with aspartame or its metabolites induced a significant elevation in the mean effect of PEaa species in SH-SY5Y cells under the conditions used in this study (aspartame: 138.5% ± 11.8% (*p* ≤ 0.001); metabolites: 144.0% ± 13.9% (*p* ≤ 0.001)) (see Figure 6C). A detailed analysis revealed that both aspartame and metabolite treatment mediated an increase above the SEM for each of the 35 PEaa species measured (see Figure 6D). In line with this, no significant difference between the two incubation conditions could be detected (*p* = 0.168; see Figure 6D). Among these common increased PEaa species were also the most common PE species of brain [39] such as C36:1, C36:4, C38:4, C38:6, and C40:6.

### 3.4. Analysis of Carnitine Species

**Figure 7:**
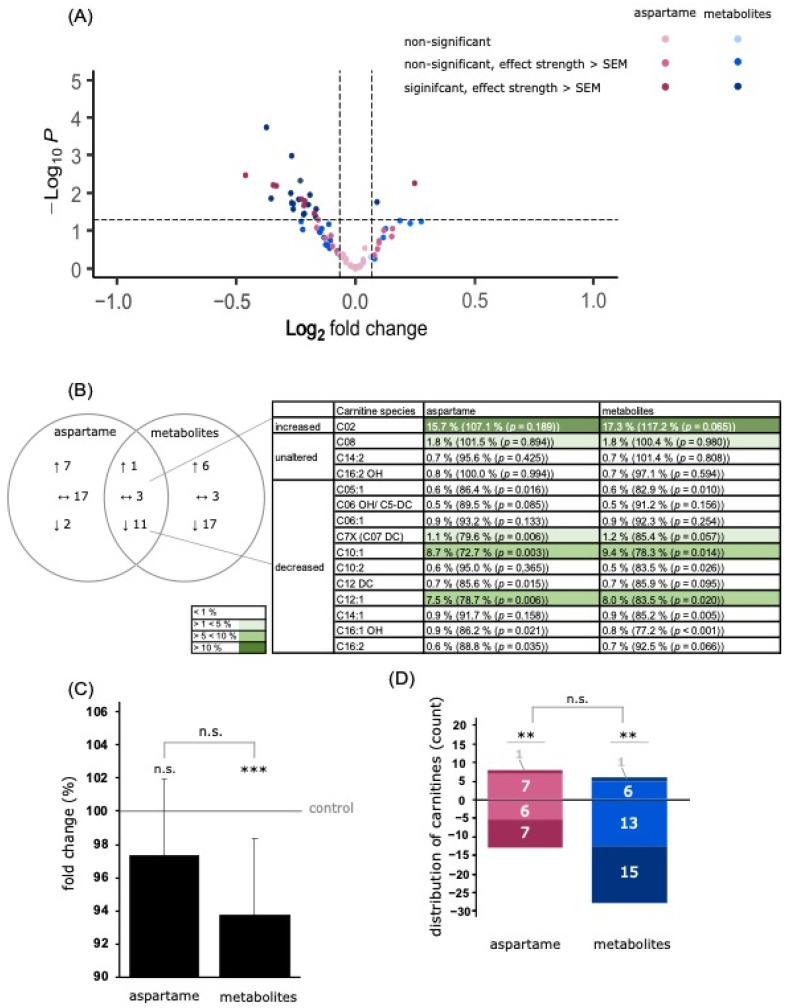
Effects of aspartame or aspartame metabolites on carnitine species. Neuroblastoma cells (SH-SY5Y) were treated as described before with control, aspartame, or aspartame metabolites for 48 h and analyzed using a semi-quantitative shotgun lipidomics approach. Each diagram represents the changes in the aspartame- or metabolite-treated cells in comparison to control-treated cells. (**A**) In the volcano plot, each carnitine species is graphically represented by a pink (aspartame treatment) or blue (aspartame metabolite treatment) dot which is plotted with its fold change (*x*-axis) against its according *p*-value (*y*-axis). The volcano plot is structured as the one in Figure 4. (**B**) Venn diagram of carnitine species after incubation of SH-SY5Y cells with aspartame or aspartame metabolites. Alterations of the same species are visible in the overlapping part. (**C**) The bar chart shows the relative fold change of all measured carnitine species after aspartame treatment compared to treatment with aspartame metabolites. Error bars represent the standard error of the mean (SEM), and the statistical significance was set as ** *p* ≤ 0.01 and *** *p* ≤ 0.001. (**D**) Distribution of carnitine species, classified by the amount of increased or decreased carnitine species. The bar chart is structured as the one in Figure 4. The applied statistical analyses are described in detail in Section 2.9 (n.s. non-significant).

Carnitines are important metabolites involved in oxidative metabolic processes such as lipid peroxidation (β-oxidation) in mitochondria. Treatment with aspartame and metabolites resulted in a slight reduction to 97.3% ± 5.4% (*p* = 0.064) and 93.7% ± 6.3% (*p* ≤ 0.001), respectively (see Figure 7C).

In this study, 41 different carnitine species were measured. After aspartame treatment, 24 carnitine species showed a left shift (see Figure 7A), of which 13 species showed an effect greater than the SEM, including seven species with a significant decrease (see Figure 7D). Seventeen species exhibited a slight right shift, of which eight were above the SEM (see Figure 7D).

Metabolite treatment led to a left shift of 29 carnitine species (see Figure 7A), of which 28 species showed a downregulation greater than the SEM, which included 15 species being significantly decreased (see Figure 7D). A right shift was detected for 12 species after metabolite treatment, including seven with an effect above the SEM (see Figure 7D).

## 4. Discussion

Aspartame is a controversial non-nutritive sweetener, as it has shown adverse effects on vertebrates in different studies. It has been linked to impaired glucose tolerance and an altered gut microbiota composition [40], as well as to changes in cognitive behavior in mice and an increased risk of certain tumors. Numerous studies have shown that aspartame causes oxidative stress in vitro and in vivo [12,13]. Cellular reactive oxidative stress is closely linked to lipid metabolism [41]. Both are known to be involved in the development of various diseases such as metabolic syndrome and diabetes type 2 [42], but also neurodegenerative diseases [43].

Aspartame is the methyl ester of a dipeptide composed of aspartic acid, phenylalanine, and methanol. Various studies in monkeys [44] and humans [45] have shown that aspartame is metabolized into its three components in the gastrointestinal lumen by non-specific esterases and peptidases. It is then absorbed through the intestinal epithelium and enters the systemic circulation. Following oral administration of aspartame at high doses (100–200 mg/kg), elevated blood levels of the individual metabolites were noted [46,47,48], but were below levels associated with toxicity.

The metabolism of aspartame after ingestion has been a point of criticism in various aspartame studies. In vitro studies using aspartame as a complete molecule have been criticized as inconclusive. Parenteral administration to subjects is considered not to be representative of oral administration [5].

In order to establish an experimental set-up as realistic as possible to oral aspartame intake in humans, we decided to perform two simultaneous but separate incubation conditions. One was incubation with aspartame as a complete molecule, the other was incubation with its three metabolites, the sum of which was equimolar to the aspartame concentration. The selected concentration of 0.08 g/L was based on the FDA recommended maximum daily dose (50 mg/kg body weight) and the resulting volume of distribution in an average-weight adult [49]. Interestingly, we found comparable effects when incubation was performed with the complete molecule or the three metabolites. This suggests that both “variants” can lead to an effect on neuronal cells, regardless of whether they reach the brain in the complete or metabolized form. However, it must be emphasized that the effects were stronger in cells treated with aspartame.

The aim of this study was to investigate aspartame-induced effects, such as oxidative stress, on lipid metabolism in a neuronal cell line, namely SH-SY5Y cells. Therefore, we examined cells treated with aspartame and aspartame metabolites by electron microscopy, measured markers of oxidative stress; performed mass spectrometric lipid analysis; and performed quantitative RT-PCR of several genes. In summary, we found indicators of increased oxidative stress, an accumulation of lipid droplets, significantly increased levels of triacylglycerol and phospholipid species, and altered expression of antioxidant enzymes and mitophagy-mediating signaling proteins.

Regarding oxidative stress, we found an impaired mitochondrial morphology, significantly increased APF levels, and upregulated gene expression of compensatory antioxidant enzymes (SOD1 and SOD2). This is consistent with the literature as there are numerous studies showing that aspartame causes oxidative stress in the brain [12,13,50] and in other tissues such as the kidney [51].

Oxidative stress has been linked to several neurodegenerative diseases. In the context of AD, ROS are thought to be a pathophysiological hallmark beside the formation of β-amyloid plaques and neurofibrillary tangles [52]. Mitochondrial dysfunction is already present in the early stages of AD [53]. Compared to control subjects, several abnormalities have been found in the hippocampus of AD patient brains, such as a reduced amount of mitochondria and increased markers of mitochondrial damage (8-hydroxyguanosine and nitrotyrosine) [54]. Furthermore, the so-called mitochondrial cascade hypothesis describes the link between a loss of mitochondrial function and favored Aβ accumulation [22]. Conversely, in vivo data also showed how Aβ accumulation leads to mitochondrial dysfunction, ROS production, and synaptic degeneration [55]. Consequently, influencing factors that increase oxidative stress in Alzheimer’s disease patients could potentially lead to an aggravation of mitochondrial dysfunction, Aβ accumulation, and cognitive function.

Parkinson’s disease is also associated with the generation of reactive oxygen species. Several studies have reported increased markers of oxidative stress in the substantia nigra, such as levels of oxidized DNA [56], proteins, and lipids [57]. In addition, Parkinson’s disease is associated with impaired mitophagy [58]. Signaling proteins such as PINK1 and Parkin are mutated in sporadic Parkinson’s disease, leading to an accumulation of dysfunctional mitochondria [59]. In addition, the neurons of Parkinson’s disease patients are more susceptible to mitochondrial dysfunction than those of individuals not suffering from this disease [60]. In our study, we found that treatment with aspartame or metabolites resulted in mitochondrial impairment and increased ROS markers. Additionally, gene expression of the mitophagy mediator PINK1 was significantly increased. However, this effect was mainly observed after treatment of cells with aspartame as a total molecule.

Furthermore, aspartame treatment dose-dependently affected gene expression of SOD1. SH-SY5Y cells incubated with a 10 times lower concentration of aspartame (27.17 µM compared to 271.7 µM) exhibited a significant reduction in SOD1 gene expression to 85.9% ± 4.3% (*p* = 0.0389), whereas those incubated with a 10 times higher concentration (2717 µM compared to 271.7 µM) exhibited a significant elevation in SOD1 gene expression to 115.3% ± 6.5% (*p* = 0.0471) (see Figure S3).

Neurodegenerative diseases such as Alzheimer’s and Parkinson’s are per se associated with oxidative stress and poorly functioning compensatory mechanisms. Any further stressor—possibly including the administration of aspartame—could therefore exacerbate existing neurodegeneration. However, even in healthy people, cerebral oxidative stress is associated with accelerated ageing processes [61]. Furthermore, early oxidative stress in the brains of adolescents is discussed as predisposing mental disorders [62].

In addition, ROS have an important influence on the lipid metabolism. In our study, we found an accumulation of lipid droplets in cells treated with aspartame and metabolites compared to controls. Lipid droplets (LDs) are a physiological component of cells. In the past, LDs were mainly known as important fat stores. However, there is strong evidence that they are involved in the regulation of the cellular metabolism and represent a compensatory mechanism for cellular stress. Several studies have demonstrated the accumulation of lipid droplets and the synthesis of triacylglycerol in cells with increased cellular stress and nutrient deprivation [63]. Cabodevilla et al. found that fatty acid synthesis and LD formation in starving cells is an important mechanism for cell survival, even in neuronal cells [64]. In cells exposed to oxidative stress, the accumulation of LD serves to protect the phospholipid membrane from peroxidation and maintain its saturation [65,66]. Consistent with these findings, LDs are known to interact with mitochondria by forming (proteinaceous) contact sites. This is possible due to the unique phospholipid monolayer of lipid droplets [67,68]. The resulting “membrane bridges” allow bidirectional exchange of fatty acids between LDs and mitochondria; the degradation of TAGs from the inside of the LD releases fatty acids that can be metabolized via mitochondrial β-oxidation and other pathways to produce energy (ATP) for the cell. Conversely, mitochondria release their fatty acids to the LD to protect themselves from lipotoxicity. Nguyen et al. were able to show how potentially lipotoxic fatty acids are transferred from the mitochondria to LDs in the case of nutrient abundance and consistent mitophagy [69]. The major envelope lipids of the LD are phosphatidylcholine and phosphatidylethanolamine species; the major core lipids are neutral lipids such as triacylglycerol and cholesterol species. In our study, we found an accumulation of lipid droplets in cells treated with aspartame and metabolites compared to controls. A mass spectrometric analysis of the different lipid species revealed a significant increase in phospholipids (PCaa and PEaa) and triacylglycerol species (see Figure 4, Figure 5 and Figure 6). We could see comparable effect strengths for aspartame and metabolite treatment for most of the lipid subspecies. When ranking the lipid subspecies of cells treated with metabolites, starting with the largest effect strength in descending order, it can be clearly seen that there are comparable effects for the respective lipid subspecies of the aspartame-treated cells (see Figure S4A,B).

Only a few lipid subspecies such as TAG C52:0 and TAG C54:4 showed a differing tendency between metabolites versus aspartame treatment (see Figure S4C,D).

Carnitine species were seen to be slightly decreased, which is consistent with the literature. Additionally, here we saw related effects between both treatment conditions, except for a few carnitine subspecies such as carnitine C05 OH (see Figure S4).

Moreover, the observed effects on oxidative stress and lipid homeostasis seem to require aspartame or its three metabolites in combination. When analyzing the treatment of cells with one single metabolite such as phenylalanine, we detected only mild effects in comparison to control cells. Gene expressions of the mitochondrial markers *FIS1* and *PINK1* were slightly elevated to 104.4% (*p* = 0.396) and 102.4% (*p* = 0.710), respectively, after aspartame treatment. Expression of *SOD1* and *SOD2* was at 101.8% (*p* = 0.804) and 108.2% (*p* = 0.338), respectively (see Figure S2A). In addition, mass spectrometric analyses of the lipid species PCaa (see Figure S2B–D) and TAG (see Figure S2E–G) revealed only moderate effects after phenylalanine treatment.

Lastly, it has to be mentioned that although we found significant changes in lipid homeostasis, mitochondrial damage, and oxidative stress in the presence of aspartame or aspartame metabolites, one has to mention that our analysis is based on a cell-culture study that has to be proven in vivo. For example, it must be considered that aspartame has to cross several “barriers” after oral intake before it reaches the brain. There is the intestinal epithelium—where metabolization may take place—and the blood–brain barrier. Interestingly, Shil et al. found that aspartame increases the permeability of intestinal epithelial cells by down-regulating the expression of the transmembrane protein claudin-3, which is part of tight junctions [70]. These epithelial tight junctions are required to separate the intestinal lumen from the bloodstream and regulate the uptake of micro- and macro-nutrients. Aspartame-induced impairment of tight junctions could affect the integrity of the gut. Furthermore, these findings seem relevant considering that diseases such as celiac disease, irritable bowel syndrome [71], type I diabetes, and multiple sclerosis [72] are per se associated with a disrupted epithelial barrier, whereby exotoxins may contribute to disease pathogenesis [73]. This effect could be enhanced by aspartame-induced dysfunctional tight junctions and a consequently facilitated influx of toxic substances, but also by the non-nutritive sweetener itself. However, these in vitro results still need to be confirmed in vivo. In addition to the barrier of the intestinal epithelium, the blood–brain barrier must also be overcome by all kinds of substances. Inside the brain vessels is a highly selective, permeable endothelial membrane (with tight junctions) surrounded by vascular smooth muscle cells, pericytes, and end feet of astrocytes [74]. The BBB is known to be disrupted in various neurodegenerative diseases such as Alzheimer’s or Parkinson’s disease [75]. This in turn favors the exposure of neurons to potentially toxic metabolites, leading to acceleration or exacerbation of these diseases. It can be concluded that there are certain vulnerable groups of people who are particularly susceptible to aspartame-mediated effects on neuronal cells. This makes it all the more necessary to investigate the effects of aspartame and possible changes in the brain metabolism, which must be addressed in further studies.

## 5. Conclusions

In conclusion, our results show that both aspartame and also its metabolites affect lipid composition in neuroblastoma cells, accompanied by an increase in oxidative stress and mitochondrial changes. Under healthy conditions, where the blood–brain barrier and the intestinal barrier are intact, aspartame is known to be mainly metabolized into its three metabolites, aspartic acid, phenylalanine, and methanol, and will not be able to affect neurons as an unmetabolized molecule. Therefore, it is even more important that the metabolites also show weaker but comparable effects compared to aspartame. Elevated levels of triacylglycerides, lipid droplet formation, and oxidative stress play an important role in several diseases, including neurodegenerative diseases. Although these results must be proven in clinical studies, chronic administration of aspartame or intake of higher-than-recommended dosages should be viewed with caution.

## Figures and Tables

**Table 1:** Internal standards for analysis of different lipid species.

Name	Provider
Phosphatidylcholine (06:0 PC (DHPC))	Avanti Polar Lipids (850305P)
Carnitines (octanoyl- and palmitoyl-L-carnitine d3)	Supelco Analytical (53230, 55107)
Triacylglycerides (15:0-18:1(d7)-15:0)	Avanti Polar Lipids (330709)

**Table 2:** Parameters for the detection of different lipid species.

Parameter	
Curtain gas (CUR)	20 psi
Temperature (TEM)	200 °C
Ion source gas 1 (GS1)	40 psi
Ion source gas 2 (GS2)	50 psi
Interface heater (ihe)	On
Collisionally activated dissociation gas (CAD)	Medium
Ion spray voltage (IS)	5500 V
Entrance potential (EP)	10 V
Collision cell exit potential (cxp)	15 V

**Table 3:** Forward and reverse primers used for analysis of gene expression.

Gene	Forward Primer (5′-3′)	Reverse Primer (5′-3′)
*HPRT*	TGACACTGGCAAAACAATGCA	GGTCCTTTTCACCAGCAAGCT
*FIS1*	TACGTCCGCGGGTTGCT	CCAGTTCCTTGGCCTGGTT
*PINK1*	GGACGCTGTTCCTCGTTA	ATCTGCGATCACCAGCCA
*SOD1*	CAGCAGGCTGTACCAGTGC	ACATTGCCCAAGTCTCCAAC
*SOD2*	TACGTGAACAACCTGAACGT	CAAGCCATGTATCTTTCAGTTA

## Data Availability

Not applicable.

## Supplementary Materials

The following supporting information can be downloaded at: https://www.mdpi.com/article/10.3390/nu15061467/s1, Figure S1: Structural formula showing the enzymatic degradation of one molecule of aspartame into its three metabolites aspartic acid, phenylalanine and methanol in simplified form; Figure S2: Effects of phenylalanine on gene expression and selected lipid species (PCaa and TAG). Neuroblastoma cells (SH-SY5Y) were treated with control or phenylalanine (271.7 μM) for 48 h. Figure S3: Gene expression of *SOD1* in dependence of different doses of aspartame. Figure S4: Comparison of the relative fold changes of the upregulated (A) and downregulated (B) lipid species in SH-SY5Y cells treated with metabolites (blue bars) or aspartame (pink bars) in a bar chart sorted by effect strength of metabolites.
