# Supplementary material for: Aspartame and Its Metabolites Cause Oxidative Stress and Mitochondrial and Lipid Alterations in SH-SY5Y Cells
Source: Nutrients. 2023 Mar 18;15(6):1467. doi: 10.3390/nu15061467 (PMC10053704; doi:10.3390/nu15061467)
Supplement: Supplementary file 1 [file nutrients-15-01467-s001.zip › nutrients-2254706-supplementary.pdf]

Supplementary materials

Aspartame and Its Metabolites Cause Oxidative Stress and Mitochondrial and Lipid Alterations in SH-SY5Y Cells

Lea Victoria Griebsch [1,†], Elena Leoni Theiss [1,†], Daniel Janitschke [1], Vincent Konrad Johannes Erhardt [1], Tobias Erhardt [2], Elodie Christiane Haas [1], Konstantin Nicolas Kuppler [1], Juliane Radermacher [1], Oliver Walzer [1], Anna Andrea Lauer [1,3], Veronika Matschke [4], Tobias Hartmann [5], Marcus Otto Walter Grimm [1,3,5,*,‡] and Heike Sabine Grimm [1,3,*,‡]

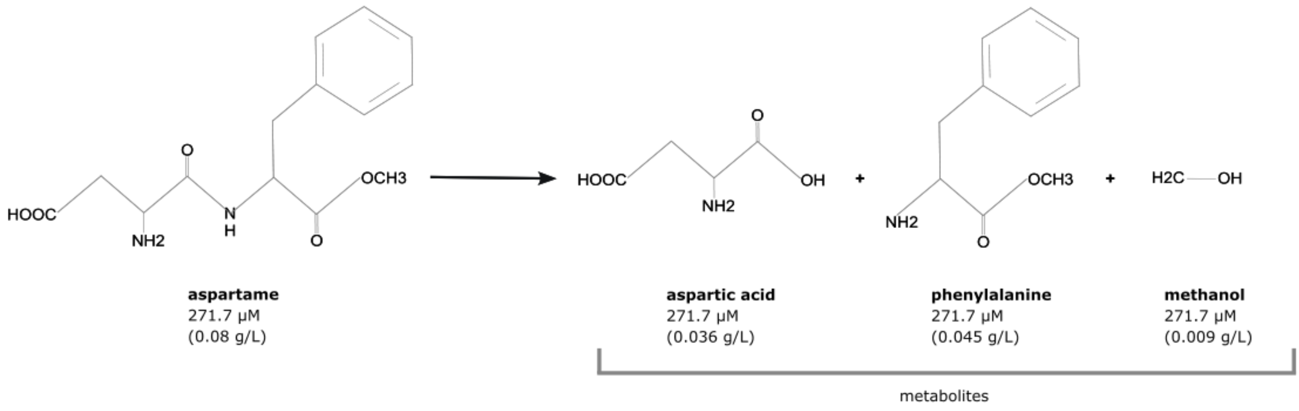

Figure S1 Structural formula showing the enzymatic degradation of one molecule of aspartame into its three metabolites aspartic acid, phenylalanine and methanol in simplified form.

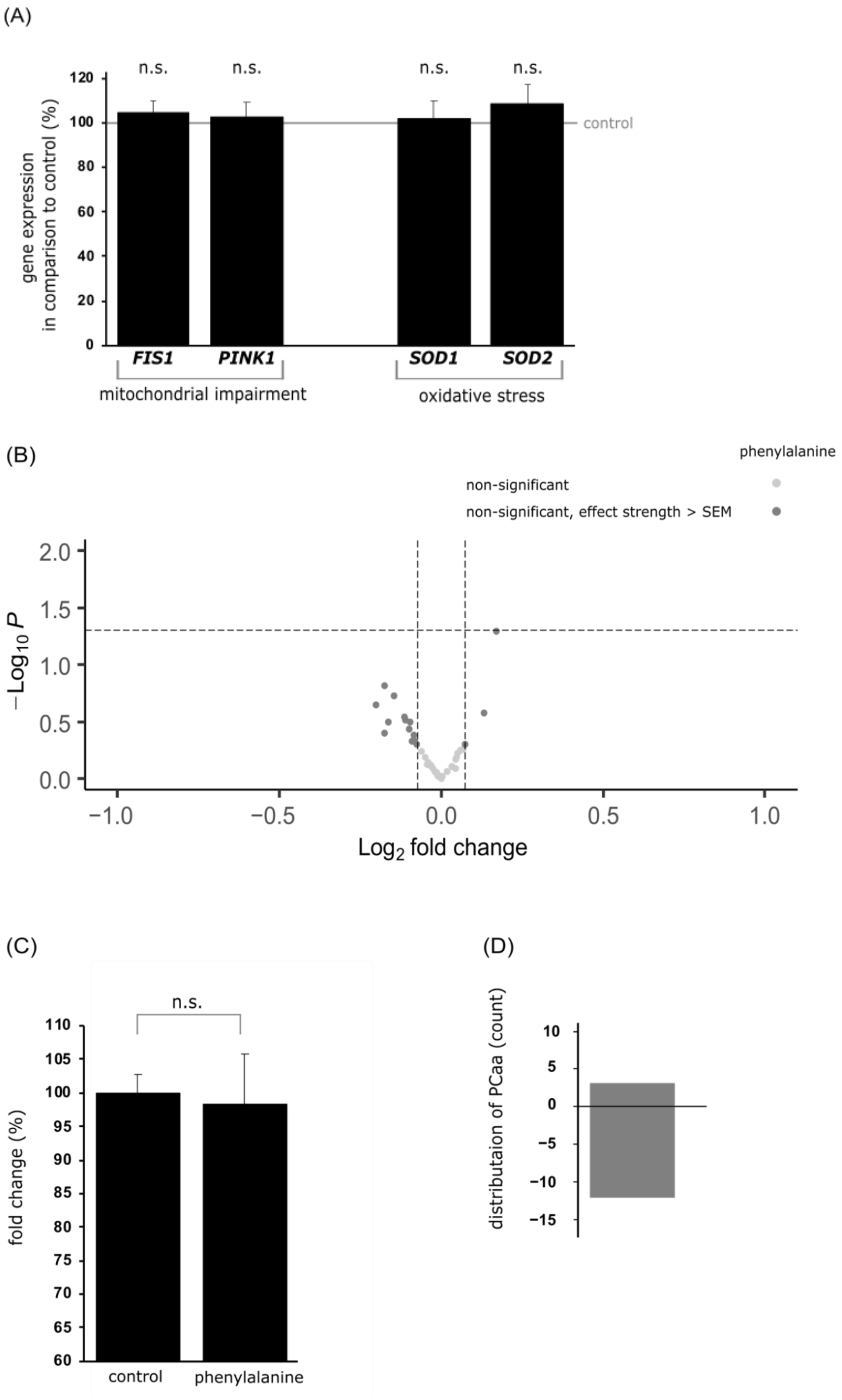

This figure continues the next page.

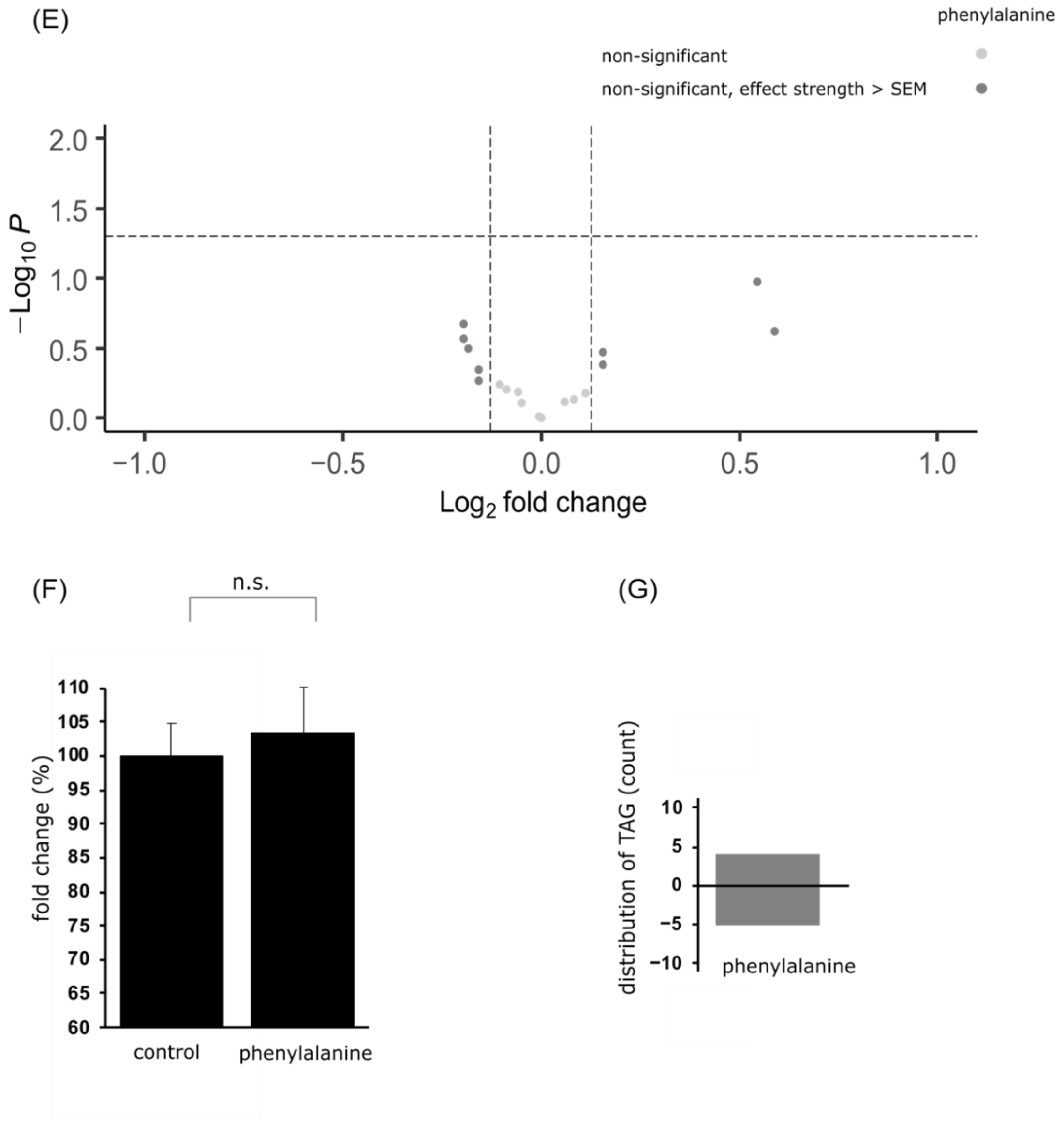

Figure S2 Effects of phenylalanine on gene expression and selected lipid species (PCaa and TAG). Neuroblastoma cells (SH-SY5Y) were treated with control or phenylalanine (271.7 µM) for 48 hours. (**A**) Levels of gene expression of *FIS1, PINK1, SOD1* and *SOD2* were detected and are illustrated in a bar chart. Error bars represent the standard error of the mean (SEM). (**B**)-(**D**) Effects of phenylalanine on phosphatidylcholine (PCaa) species using a semi-quantitative shotgun lipidomics approach. Each diagram represents the changes of phenylalanine in comparison to control-treated cells. (B) In the volcano plot each PCaa species is graphically represented by a dot which is plotted with its fold change (x-axis) against its according p-value (y-axis). Light grey dots represent no significant changes. Medium grey dots represent a fold change which is greater than the mean standard error of the mean (SEM). (C) The bar chart shows the relative fold change of all measured PCaa species after phenylalanine treatment compared to control treatment. Error bars represent the standard error of the mean (SEM). (D) Distribution of PCaa species, classified by the amount of increased or decreased PCaa species with a fold change which is greater than the mean standard error of the mean (SEM). (**E**)-(**G**) Effects of phenylalanine on triacylglycerol (TAG) species using a semi-quantitative shotgun lipidomics approach. Each diagram represents the changes of the phenylalanine in comparison to control-treated cells. A detailed description of the structure of the volcano plot and the bar charts was previously given (see B-D).

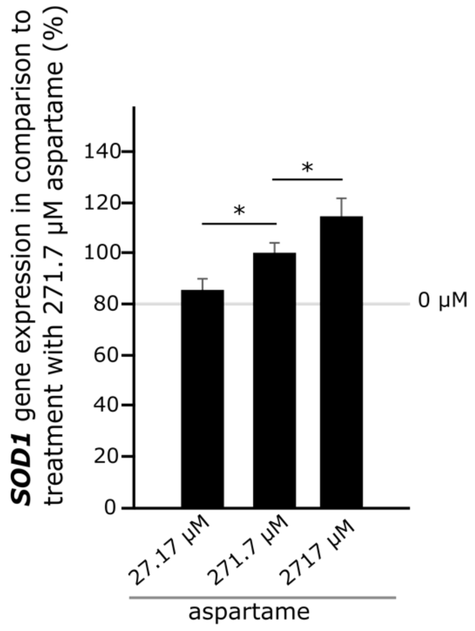

Figure S3 Gene expression of *SOD1* in dependence of different doses of aspartame. Neuroblastoma cells (SH-SY5Y) were treated with 27.17 µM, 271.7 µM or 2717 µM aspartame for 48 hours and levels of gene expression of *SOD1* were detected via quantitative RT-PCR. The dosages decreased or increased by a factor of 10 (27.17 µM and 2717 µM) were set as relative to dosage 271.7 µM, which was used throughout this study, to examine if aspartame dose-dependently causes oxidative stress. * $p \leq 0.05$

(A)

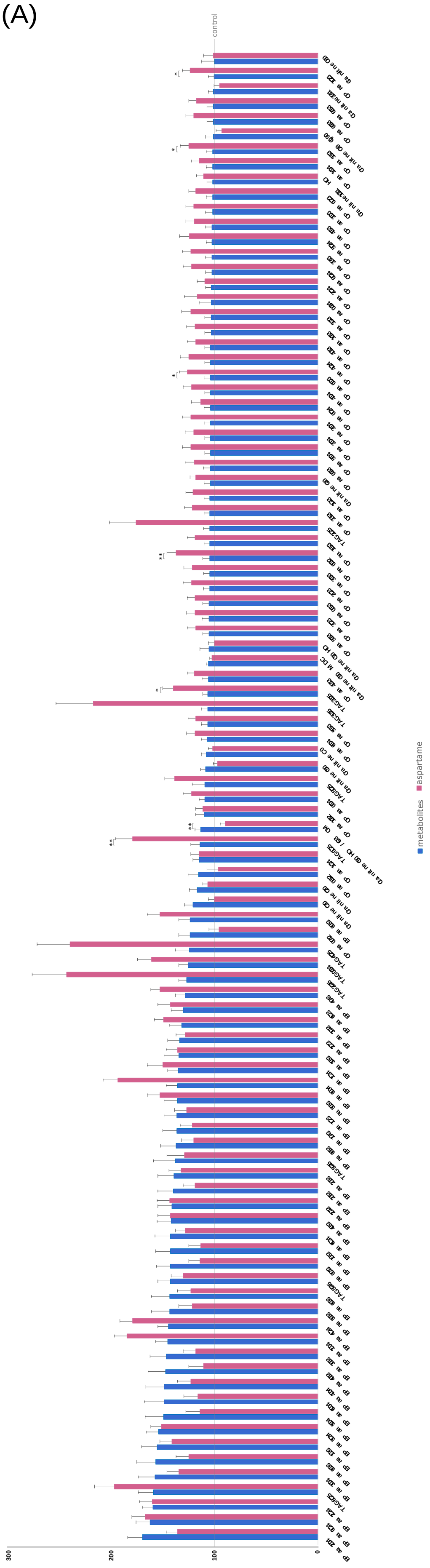

(B)

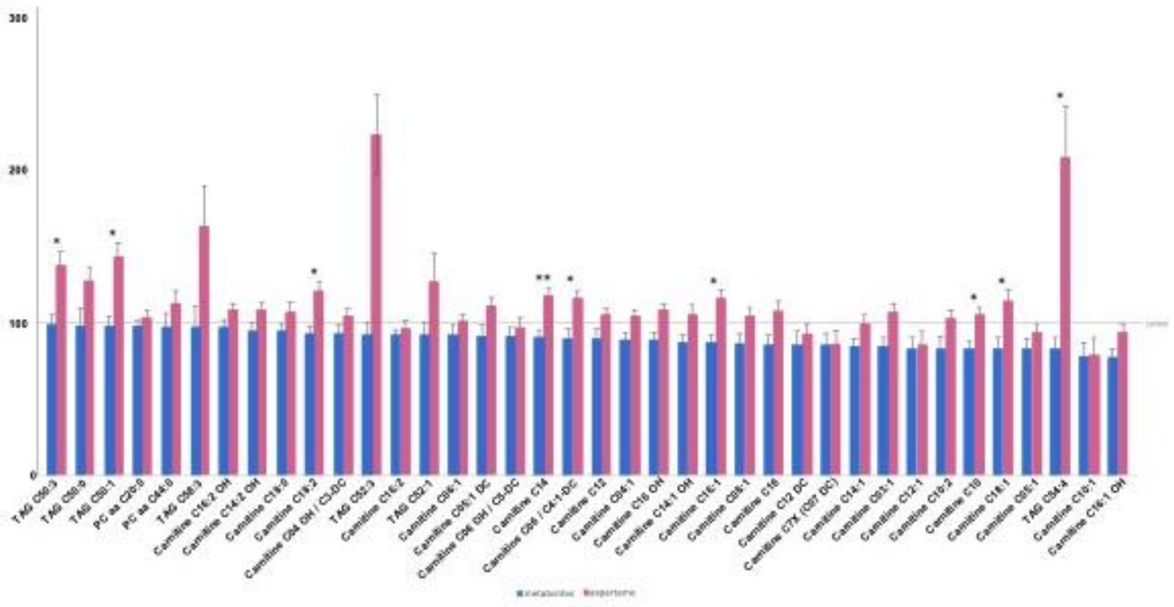

(C)

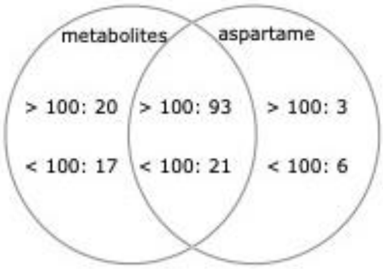

(D)

Lipid subspecies	metabolites (vs. control in %)	aspartame (vs. control in %)	significance aspartame vs. metabolites
TAG C52:0	114.7	180.2	0.006
Carnitine C05 OH / C3-DC-M	113.8	89.8	0.002
TAG C50:2	106.7	140.7	0.013
PC aa C28:0	104.9	137.8	0.005
PC aa C30:0	104.1	127.0	0.031
PC aa C36:3	101.9	125.4	0.040
PC aa 32:1	100.6	124,0	0.029
TAG C50:3	98.7	127.2	0.014
TAG C50:1	98.5	132.5	0.011
Carnitine C18:2	93.0	111.4	0.011
Carnitine C14	90.6	108.8	0.007
Carnitine C06 / C4:1-DC	90.3	107.1	0.040
Carnitine C16:1	87.1	106.7	0.013
Carnitine C10	83.2	97.4	0.039
Carnitine C18:1	83.1	105.7	0.011
TAG C54:4	82.9	192.2	0.017

Figure S4 Comparison of the relative fold changes of the upregulated (**A**) and downregulated (**B**) lipid species in SH-SY5Y cells treated with metabolites (blue bars) or aspartame (pink bars) in a bar chart sorted by effect strength of metabolites. Error bars represent the standard error of the mean (SEM). Statistical significance between aspartame versus metabolites was set as * $p \leq 0.05$ and ** $p \leq 0.01$. (**C**) Venn diagram summarizing the number of exclusively or uniformly de- or increased lipid species in SH-SY5Y cells treated with aspartame or metabolites. (**D**) The table lists those lipid species that differ significantly in effect strength between treatment with aspartame or metabolites.
